# Diabetes Mellitus Is an Independent Predictor of Short-Term Mortality in Critically Ill ICU Patients

**DOI:** 10.3390/healthcare14040452

**Published:** 2026-02-11

**Authors:** Mădălina Diana Daina (Fehér), Codrin Dan Nicolae Ilea, Cosmin Mihai Vesa, Alina Cristiana Venter, Adriana Vladu, Timea Claudia Ghitea, László Fehér, Cristian Marius Daina

**Affiliations:** 1 Doctoral School of Biomedical Sciences, Faculty of Medicine and Pharmacy, University of Oradea, 410081 Oradea, Romania; 2 Department of Psycho-Neurosciences and Recovery, Faculty of Medicine and Pharmacy, University of Oradea, 410081 Oradea, Romania; cristi_daina@yahoo.co.uk; 3 Department of Preclinical Disciplines, Faculty of Medicine and Pharmacy, University of Oradea, 410081 Oradea, Romania; 4 Department of Morphological Disciplines, Faculty of Medicine and Pharmacy, University of Oradea, 410073 Oradea, Romania; aventer@uoradea.ro; 5 Department of Surgical Disciplines, Faculty of Medicine and Pharmacy, University of Oradea, 410081 Oradea, Romania; 6 Pharmacy Department, Faculty of Medicine and Pharmacy, University of Oradea, 410081 Oradea, Romania

**Keywords:** critical patient, diabetes mellitus, ICU ward, risk of death

## Abstract

**Highlights:**

**What are the main findings?**

- Diabetes mellitus was present in nearly one-third of critically ill ICU patients and was associated with significantly higher short-term mortality.
- After adjustment for major clinical confounders, diabetes mellitus remained an independent predictor of ICU mortality.

**What are the implications of the main findings?**

- Diabetes status should be considered an important prognostic factor during early ICU risk assessment.
- Critically ill patients with diabetes may benefit from closer monitoring and individualized management strategies in intensive care settings.

**Abstract:**

**Background**: Diabetes mellitus (DM) is frequently encountered in critically ill patients and has been associated with poor outcomes. However, its independent impact on short-term mortality in heterogeneous ICU populations remains unclear. **Objectives**: To evaluate whether diabetes mellitus is an independent risk factor for intensive care unit (ICU) mortality in critically ill adult patients. **Methods**: We conducted a single-center retrospective observational study including adult patients admitted to the ICU between January and December 2024. Patients were stratified according to the presence or absence of diabetes mellitus. Demographic data, major clinical variables, and ICU outcomes were analyzed. The primary endpoint was ICU mortality. A multivariate logistic regression model was used to identify independent predictors of death. **Results**: A total of 1344 patients were included, of whom 435 (32.4%) had diabetes mellitus. ICU mortality was significantly higher in patients with DM compared to non-diabetic patients (54.9% vs. 46.3%, *p* = 0.004). After adjustment for age, sex, sepsis, acute kidney injury, and mechanical ventilation, diabetes mellitus remained independently associated with an increased risk of ICU death. **Conclusions**: Diabetes mellitus is an independent predictor of short-term mortality in critically ill ICU patients. Early identification and risk stratification of diabetic patients may improve clinical management and outcomes in intensive care settings.

## 1. Introduction

Diabetes mellitus (DM) is one of the most prevalent chronic diseases worldwide and represents a growing public health challenge. Its global burden continues to increase as a result of population aging, sedentary lifestyles, and the rising prevalence of obesity and cardiometabolic disorders. Consequently, diabetes mellitus is increasingly encountered among critically ill patients admitted to intensive care units (ICUs) [1,2,3].

In the critical care setting, patients with DM often present with a complex clinical profile characterized by multiple comorbidities, metabolic dysregulation, and reduced physiological reserve. These factors may contribute to a higher vulnerability during acute illness and may influence short-term outcomes, including survival. However, the independent role of diabetes mellitus in determining ICU mortality remains incompletely understood [4,5,6,7].

Diabetes mellitus is also characterized by chronic low-grade inflammation and immune dysregulation, which may amplify the systemic inflammatory response during critical illness. Inflammation plays a central role in the pathophysiology of organ failure and mortality in ICU patients. Therefore, the coexistence of diabetes and acute critical illness may create a pro-inflammatory milieu that contributes to worse outcomes [8].

Previous studies evaluating the impact of diabetes on ICU mortality have reported inconsistent findings. While some investigations suggest that diabetes is associated with increased short-term mortality, others have found comparable or even lower mortality rates among diabetic patients compared to non-diabetic patients. These discrepancies may be explained by differences in study populations, ICU case-mix, severity of illness, and methodological approaches [9,10,11,12].

Given this variability and the limited availability of real-world data derived from heterogeneous ICU populations, further evaluation of the prognostic role of diabetes mellitus is warranted. Therefore, the present study aimed to assess the association between diabetes mellitus and short-term mortality in critically ill adult patients admitted to a tertiary care intensive care unit.

Although the association between diabetes and ICU outcomes has been previously explored, contemporary real-world data from Eastern European ICU settings remain limited. Our study provides updated evidence from a large tertiary-care cohort reflecting current ICU practice and the increasing prevalence of diabetes among critically ill patients. This context-specific evidence may help refine local risk stratification and resource planning [13].

Therefore, the aim of this study was to evaluate whether diabetes mellitus is independently associated with short-term mortality in critically ill adult ICU patients. To address this objective, we conducted a retrospective observational study in a tertiary care intensive care unit, analyzing demographic and clinical characteristics as well as ICU outcomes in critically ill adult patients with and without diabetes mellitus.

## 2. Materials and Methods

### 2.1. Study Design and Population

We conducted a retrospective observational study in the Intensive Care Unit of the Bihor County Emergency Clinical Hospital, including all adult patients admitted between 1 January and 31 December 2024.

**Figure 1:**
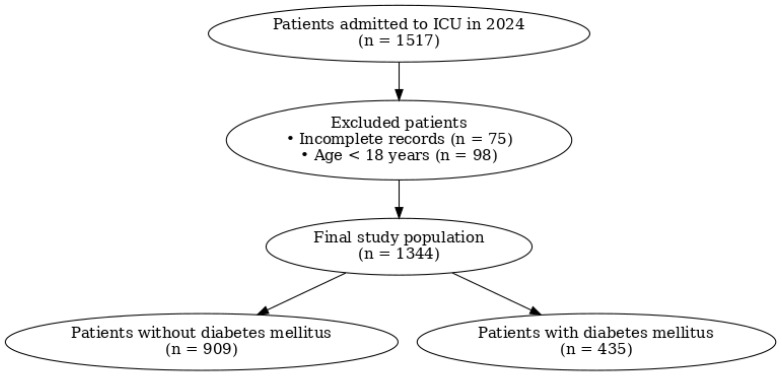
Flowchart of patient selection. During the study period, 1517 patients were admitted to the ICU. After exclusion of patients with incomplete medical records (n = 75) and patients younger than 18 years (n = 98), a total of 1344 adult patients were included in the final analysis. Of these, 909 patients had no diabetes mellitus, while 435 patients were diagnosed with diabetes mellitus.

During the study period, 1517 patients were admitted to the ICU from medical wards, surgical wards (both postoperative and non-operative cases), or transferred from other hospitals. Patients younger than 18 years, those with incomplete medical records, patients with secondary forms of diabetes mellitus (drug-induced or related to endocrinopathies), and patients with gestational diabetes were excluded. After applying the exclusion criteria, a total of 1344 adult patients were included in the final analysis (Figure 1). The ICU serves a mixed medical–surgical population, including medical, postoperative, and emergency admissions, reflecting a heterogeneous tertiary-care ICU setting.

Diabetes mellitus was defined based on a documented medical history of diabetes, ongoing antidiabetic therapy, or diagnostic glycemic criteria including HbA1c ≥ 6.5%. Patients presenting with transient hyperglycemia without elevated HbA1c were not classified as diabetic, thereby reducing misclassification due to stress hyperglycemia.

Differentiation between type 1 and type 2 diabetes and precise disease duration were not consistently available in the medical records. Therefore, diabetes was analyzed as a binary variable.

Organ-support therapies such as mechanical ventilation, vasopressor use, and renal dysfunction were used as proxies for severity, as they represent core components of validated ICU scores.

Because validated ICU severity scores (e.g., APACHE II, SOFA, SAPS II) could not be calculated from the available retrospective data, illness severity was partially accounted for using clinically relevant organ-support variables. Specifically, the need for mechanical ventilation, inotropic/vasopressor support, and renal dysfunction were included as proxies for organ failure. These variables represent core components of established ICU severity scores and are recognized indicators of critical illness severity.

### 2.2. Data Collection

For each patient, the following data were collected from electronic medical records: demographic characteristics (age and sex), diabetes mellitus status, primary reason for ICU admission, and clinical outcome.

Additional clinical variables included major acute conditions and interventions relevant to critical illness, such as sepsis, acute kidney injury, and the need for mechanical ventilation. The primary outcome of interest was mortality during ICU hospitalization.

In addition to overall ICU mortality, 7-day and 28-day mortality were calculated based on the interval between ICU admission and death.

### 2.3. Statistical Analysis

Continuous variables were expressed as mean ± standard deviation, while categorical variables were reported as absolute numbers and percentages. Comparisons between patients with and without diabetes mellitus were performed using Student’s *t*-test for continuous variables and the chi-square test or Fisher’s exact test, as appropriate, for categorical variables.

To identify independent predictors of ICU mortality, a multivariate logistic regression model was constructed. Variables were selected a priori based on clinical relevance and established associations with mortality in critically ill patients, including age, sex, sepsis, acute kidney injury, mechanical ventilation, and diabetes mellitus status. Results were reported as adjusted odds ratios (ORs) with 95% confidence intervals (CIs).

Model performance was assessed using discrimination and calibration metrics, including the area under the receiver operating characteristic curve (AUC/ROC), the Hosmer–Lemeshow goodness-of-fit test, and the Brier score.

All statistical analyses were performed using IBM SPSS Statistics for Windows, version 30.0 (IBM Corp., Armonk, NY, USA). A two-sided *p*-value < 0.05 was considered statistically significant.

### 2.4. Ethical Considerations

The study was conducted in accordance with the Declaration of Helsinki and approved by the Ethics Committee of the Bihor County Emergency Clinical Hospital (Approval No. 36976/23.10.2023) and the Ethics Council of the same institution (Opinion No. 36746/20.10.2023). Due to the retrospective nature of the study, the requirement for informed consent was waived. All data were anonymized prior to analysis to ensure patient confidentiality.

## 3. Results

### 3.1. Study Population and Baseline Characteristics

During the study period, a total of 1344 adult patients admitted to the intensive care unit were included in the final analysis (Figure 1). The age of the patients ranged from 19 to 96 years, with a mean age of 67.95 ± 13.14 years and a median age of 70 years.

The largest proportion of patients belonged to the 60–79 years age group, accounting for 58.56% of the total cohort, with a peak observed in the 60–69 years interval (409 patients, 30.4%). Younger patients were underrepresented, with only 1.64% aged 18–29 years and 0.97% aged over 90 years.

**Figure 2:**
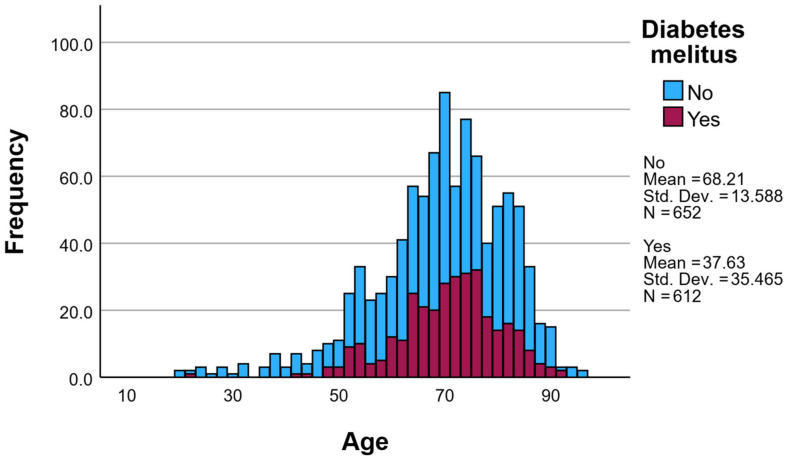
Age distribution of ICU patients according to diabetes mellitus status. The histogram illustrates the age distribution of critically ill patients admitted to the ICU, stratified by the presence or absence of diabetes mellitus. Patients without diabetes mellitus are represented in blue, while patients with diabetes mellitus are shown in burgundy. Diabetic patients were generally older compared to non-diabetic patients, with a higher frequency observed in the seventh and eighth decades of life. Mean age, standard deviation, and sample size are displayed for each group.

Diabetes mellitus (DM) was identified in 435 patients (32.37%), while 909 patients (67.63%) had no documented diagnosis of diabetes. The prevalence of diabetes increased with age, reaching its highest value in patients aged 70–79 years (43.39%), followed by those aged 60–69 years (34.23%) (Figure 2). In patients younger than 40 years, diabetes was rare.

Patients with diabetes were significantly older than non-diabetic patients, with a mean age of 69.62 ± 10.26 years compared to 67.16 ± 14.26 years in the non-diabetic group (*p* < 0.001). The median age was 71 years in patients with DM versus 69 years in those without DM (Table 1).

Regarding sex distribution, diabetic patients were predominantly female (248 women, 57.0%), whereas the non-diabetic group showed a male predominance (491 men, 54.0%). This difference was statistically significant (*p* = 0.0002).

Concerning the reason for ICU admission, patients with diabetes were significantly more likely to be admitted for medical conditions compared to non-diabetic patients (73.79% vs. 64.36%, *p* = 0.0007). In contrast, surgical admissions were more frequent among patients without diabetes (35.64% vs. 26.21%, Table 2).

Overall, the study population represents a heterogeneous cohort of critically ill adults, with a substantial proportion of elderly patients and a high prevalence of diabetes mellitus, underscoring the clinical relevance of evaluating diabetes as a prognostic factor in the ICU setting.

### 3.2. ICU Mortality

Overall ICU mortality in the study population was 49.1% (660 deaths out of 1344 patients). Mortality differed significantly according to diabetes mellitus status.

Patients with diabetes mellitus had a significantly higher ICU mortality rate compared to non-diabetic patients (54.9% vs. 46.3%, *p* = 0.004). This difference remained clinically relevant and could not be explained solely by age distribution or admission type.

The proportion of deaths was consistently higher among patients with diabetes, indicating an increased short-term risk of adverse outcomes during critical illness. These findings support the hypothesis that diabetes mellitus is associated with a higher risk of mortality in the ICU setting.

Diabetes mellitus was associated with higher 28-day mortality compared to non-diabetic patients (52.8% vs. 44.3%, *p* = 0.005), while the difference in 7-day mortality did not reach statistical significance (32.6% vs. 28.9%, *p* = 0.18).

### 3.3. Multivariate Logistic Regression Analysis

To identify independent predictors of ICU mortality, a multivariate logistic regression model was constructed including clinically relevant covariates selected a priori: age, sex, sepsis, acute kidney injury, mechanical ventilation, and diabetes mellitus status, presented in Table 3. The dependent variable was death during ICU hospitalization.

After adjustment for these covariates, diabetes mellitus remained independently associated with increased ICU mortality (OR = 1.47, 95% CI 1.09–1.98, *p* = 0.011). Mechanical ventilation showed the strongest association with ICU death (OR = 49.98, 95% CI 29.12–85.77, *p* < 0.001), while acute kidney injury was also independently associated with mortality (OR = 2.02, 95% CI 1.46–2.79, *p* < 0.001). Increasing age was associated with a small but significant increase in risk per year (OR = 1.02, 95% CI 1.01–1.03, *p* = 0.002). Sex and sepsis were retained in the model due to clinical relevance; however, they did not reach statistical significance in this adjusted analysis.

### 3.4. Model Performance

The performance of the multivariate logistic regression model was evaluated using discrimination and calibration metrics. The model demonstrated good discriminatory ability, with an area under the receiver operating characteristic curve (AUC/ROC) of 0.88 (95% CI: 0.86–0.90), indicating a good discriminatory ability between survivors and non-survivors during ICU hospitalization.

Model calibration was assessed using the Hosmer–Lemeshow goodness-of-fit test, which showed no evidence of poor fit (*p* = 0.80), suggesting good agreement between observed and predicted outcomes across risk deciles. Overall prediction accuracy was further supported by a low Brier score (0.137), indicating good probabilistic performance of the model.

Together, these results confirm that the multivariate model provides robust discrimination and adequate calibration for predicting ICU mortality in critically ill patients.

## 4. Discussion

In this retrospective cohort study of critically ill adult patients admitted to a tertiary care ICU, diabetes mellitus was associated with a significantly increased risk of short-term mortality. Importantly, this association persisted after adjustment for key demographic and clinical confounders, including age, sex, sepsis, acute kidney injury, and mechanical ventilation, confirming diabetes mellitus as an independent prognostic factor for ICU mortality.

The prevalence of diabetes mellitus in our ICU population (32.4%) is consistent with previous reports from mixed medical–surgical ICUs, reflecting the growing burden of diabetes among critically ill patients. As expected, patients with diabetes were older and more frequently admitted for medical conditions, highlighting a distinct clinical profile compared to non-diabetic patients. Despite these baseline differences, the persistence of the association between diabetes and mortality after multivariable adjustment suggests that diabetes itself contributes to increased vulnerability during critical illness, beyond age or admission type alone [6,10,14,15,16,17,18].

Notably, a prior meta-analysis [10] did not demonstrate a consistent association between diabetes and ICU mortality. Several factors may explain the discrepancy with our findings. First, that meta-analysis included studies conducted before contemporary ICU management strategies and before the widespread adoption of standardized glycemic control protocols. Second, ICU case-mix and patient profiles have evolved substantially over the past decade, with a higher burden of multimorbidity and metabolic disease in current ICU populations. Third, heterogeneity across earlier studies regarding diabetes definitions, severity adjustment, and outcome measures may have diluted potential associations. In contrast, our study used standardized diagnostic criteria, contemporary data, and multivariable adjustment for key confounders, which may provide a more updated estimate of the prognostic impact of diabetes in modern ICU practice. Nevertheless, differences in design and residual confounding should be considered when interpreting these comparisons [10,19].

Our findings align with several observational studies reporting higher ICU or in-hospital mortality among patients with diabetes, particularly in the context of acute organ dysfunction and severe infections. Although the exact mechanisms were not explored in the present analysis, the increased mortality risk observed in diabetic patients may be related to a combination of factors, including impaired immune response, reduced physiological reserve, and a higher susceptibility to acute metabolic and renal complications during critical illness. However, the present study was not designed to investigate mechanistic pathways, and such interpretations should be made with caution [4,10,17,20,21,22,23].

While the association between diabetes and ICU mortality has been previously explored, most evidence derives from older datasets or highly selected populations. Contemporary real-world data from Eastern European ICU settings remain limited. Our study provides updated evidence from a large tertiary-care cohort reflecting current ICU practices and case mix. Additionally, the relatively high prevalence of diabetes in our cohort underscores the growing clinical relevance of this issue in modern critical care. The association persisted in both early and 28-day mortality analyses, supporting the robustness of the findings.

Mechanical ventilation emerged as the strongest predictor of ICU mortality, reflecting the severity of respiratory failure rather than a modifiable risk factor. Acute kidney injury was also independently associated with death, underscoring the prognostic importance of organ dysfunction in critically ill patients. Within this clinical context, diabetes mellitus appears to act as a risk amplifier, increasing the likelihood of poor outcomes when critical illness occurs. Importantly, these results support the inclusion of diabetes status in early ICU risk assessment, alongside traditional markers of disease severity [9,24,25,26,27,28,29].

From a clinical standpoint, the identification of diabetes mellitus as an independent predictor of ICU mortality has practical implications. Early recognition of diabetic status on ICU admission may aid in risk stratification and support closer monitoring and individualized management strategies in this high-risk population. Given the increasing prevalence of diabetes worldwide, these findings are particularly relevant for contemporary intensive care practice [20,30,31,32,33,34,35].

### Limitations

This study has several limitations that should be acknowledged. First, its retrospective observational design precludes the establishment of causal relationships between diabetes mellitus and ICU mortality, and residual confounding by unmeasured variables cannot be excluded.

Second, this was a single-center study, which may limit the generalizability of the findings to ICUs with different patient case-mix, admission policies, or management protocols. Multicenter studies are needed to confirm these results across diverse healthcare systems.

Third, validated ICU severity scores (e.g., APACHE II, SOFA, SAPS II) and detailed baseline comorbidity data were not consistently available and therefore could not be included in the multivariable model. To partially address illness severity, major organ-support variables such as mechanical ventilation, vasopressor use, and renal dysfunction were used as proxies; however, these do not fully replace formal severity scores, and residual confounding related to baseline severity remains possible.

Fourth, diabetes-related characteristics—including disease duration, subtype, prior glycemic control, HbA1c levels, and pre-admission antidiabetic treatments—were not consistently available. In addition, admission glucose levels, glycemic variability, insulin therapy, and hypoglycemic events were not analyzed. These factors may influence ICU outcomes and could partially mediate the observed association between diabetes and mortality.

Fifth, data on several chronic comorbidities (e.g., cardiovascular disease, chronic liver disease, obesity, and malignancy) were incomplete, which may have contributed to residual confounding.

Finally, only short-term ICU mortality was evaluated. Long-term outcomes, including post-discharge mortality and functional recovery, were beyond the scope of this study and warrant further investigation.

## 5. Conclusions

In this retrospective cohort study of critically ill adult patients, diabetes mellitus was independently associated with increased short-term mortality in the intensive care unit. Even after adjustment for major demographic and clinical confounders, the presence of diabetes remained a significant predictor of ICU death.

These findings highlight diabetes mellitus as an important prognostic marker in critically ill patients and emphasize the need for early recognition and appropriate risk stratification upon ICU admission. Given the increasing prevalence of diabetes in aging populations, integrating diabetic status into routine ICU assessment may help refine risk stratification in critically ill patients.

Further prospective and multicenter studies are warranted to better elucidate the mechanisms underlying this association and to identify targeted strategies aimed at improving outcomes in critically ill patients with diabetes mellitus.

## Figures and Tables

**Table 1:** Demographic characteristics of the study population according to diabetes status.

Variable	Total (n = 1344)	Without DM (n = 909)	with DM (n = 435)	*p*-Value
Age, years (mean ± SD)	67.95 ± 13.14	67.16 ± 14.26	69.62 ± 10.26	<0.001
Median age, years	70	69	71	–
Female sex, n (%)	666 (49.6)	418 (46.0)	248 (57.0)	0.0002
Male sex, n (%)	678 (50.4)	491 (54.0)	187 (43.0)	–
Medical admission, n (%)	906 (67.4)	585 (64.4)	321 (73.8)	0.0007
Surgical admission, n (%)	438 (32.6)	324 (35.6)	114 (26.2)	–

SD = standard deviation, DM = diabetes melitus, n = number of patients.

**Table 2:** Type of ICU admission according to diabetes mellitus status.

Type of Admission	Total (n = 1344)	Without DM (n = 909)	with DM (n = 435)	*p*-Value
Medical admission, n (%)	906 (67.4)	585 (64.4)	321 (73.8)	0.0007
Surgical admission, n (%)	438 (32.6)	324 (35.6)	114 (26.2)	–

ICU, intensive care unit; DM, diabetes mellitus; n, number of patients.

**Table 3:** Multivariate logistic regression model for ICU mortality.

Variable	Adjusted OR	95% CI	*p*-Value
Diabetes mellitus (yes vs. no)	1.47	1.09–1.98	0.011
Age (per 1-year increase)	1.02	1.01–1.03	0.002
Sex	0.84	0.64–1.10	0.209
Sepsis	1.24	0.86–1.80	0.249
Acute kidney injury	2.02	1.46–2.79	<0.001
Mechanical ventilation	49.98	29.12–85.77	<0.001

Abbreviations: OR, odds ratio; CI, confidence interval; ICU, intensive care unit.

## Data Availability

The raw data supporting the conclusions of this article will be made available by the authors on request.

## Abbreviations

AKI	acute kidney injury;
AUC	area under the curve;
CI	confidence interval;
DM	diabetes mellitus;
HbA1c	glycated hemoglobin;
ICU	intensive care unit;
OR	odds ratio;
ROC	receiver operating characteristic;
SD	standard deviation.

## References

[1] Hossain M.J., Al-Mamun M., Islam M.R. (2024). Diabetes mellitus, the fastest growing global public health concern: Early detection should be focused. Health Sci. Rep..

[2] Pan C., Cao B., Fang H., Liu Y., Zhang S., Luo W., Wu Y. (2025). Global burden of diabetes mellitus 1990–2021: Epidemiological trends, geospatial disparities, and risk factor dynamics. Front. Endocrinol..

[3] Khan M.A.B., Hashim M.J., King J.K., Govender R.D., Mustafa H., Al Kaabi J. (2020). Epidemiology of type 2 diabetes—Global burden of disease and forecasted trends. J. Epidemiol. Glob. Health.

[4] Guan H., Tian J., Wang Y., Niu P., Zhang Y., Zhang Y., Fang X., Miao R., Yin R., Tong X. (2024). Advances in secondary prevention mechanisms of macrovascular complications in type 2 diabetes mellitus patients: A comprehensive review. Eur. J. Med. Res..

[5] Antar S.A., Ashour N.A., Sharaky M., Khattab M., Ashour N.A., Zaid R.T., Roh E.J., Elkamhawy A., Al-Karmalawy A.A. (2023). Diabetes mellitus: Classification, mediators, and complications; A gate to identify potential targets for the development of new effective treatments. Biomed. Pharmacother..

[6] Bodke H., Wagh V., Kakar G. (2023). Diabetes Mellitus and Prevalence of Other Comorbid Conditions: A Systematic Review. Cureus.

[7] Paduraru L., Vesa C.M., Popoviciu M.S., Ghitea T.C., Zaha D.C. (2025). Interrelationship between dyslipidemia and hyperuricemia in patients with uncontrolled type 2 diabetes: Clinical implications and a risk identification algorithm. Healthcare.

[8] Alexander M., Cho E., Gliozheni E., Salem Y., Cheung J., Ichii H. (2024). Pathology of diabetes-induced immune dysfunction. Int. J. Mol. Sci..

[9] Yayan J., Rasche K. (2026). Impact of diabetes mellitus on 30-day mortality and ventilation outcomes in critically ill patients with acute exacerbation of chronic obstructive pulmonary disease (AECOPD): A retrospective cohort study. Life.

[10] Siegelaar S.E., Hickmann M., Hoekstra J.B., Holleman F., DeVries J.H. (2011). The effect of diabetes on mortality in critically ill patients: A systematic review and meta-analysis. Crit. Care.

[11] Falciglia M., Freyberg R.W., Almenoff P.L., D’Alessio D.A., Render M.L. (2009). Hyperglycemia-related mortality in critically ill patients varies with admission diagnosis. Crit. Care Med..

[12] Vulciu P.A., Pilat L., Mot M.-D., Dascau V., Popa C.D., Varga N.-I., Puschita M. (2025). Tetranectin and paraoxonase 1 in patients with varying stages of heart failure: A cross-sectional analysis. Clin. Pract..

[13] Sechterberger M.K., Bosman R.J., Oudemans-van Straaten H.M., Siegelaar S.E., Hermanides J., Hoekstra J.B., De Vries J.H. (2013). The effect of diabetes mellitus on the association between measures of glycaemic control and ICU mortality: A retrospective cohort study. Crit. Care.

[14] Krinsley J.S., Roberts G., Brownlee M., Schwartz M., Preiser J.-C., Rule P., Wang Y., Bahgat J., Umpierrez G.E., Hirsch I.B. (2022). Case-control investigation of previously undiagnosed diabetes in the critically ill. J. Endocr. Soc..

[15] Pinedo-Torres I., Flores-Fernández M., Yovera-Aldana M., Gutierrez-Ortiz C., Zegarra-Lizana P., Intimayta-Escalante C., Moran-Mariños C., Alva-Diaz C., Pacheco-Barrios K. (2020). Prevalence of diabetes mellitus and its associated unfavorable outcomes in patients with acute respiratory syndromes due to coronaviruses infection: A systematic review and meta-analysis. Clin. Med. Insights Endocrinol. Diabetes.

[16] Osuagwu U.L., Xu M., Piya M.K., Agho K.E., Simmons D. (2022). Factors associated with long intensive care unit (ICU) admission among inpatients with and without diabetes in South Western Sydney public hospitals using the New South Wales admission patient data collection (2014–2017). BMC Endocr. Disord..

[17] Afkarian M., Sachs M.C., Kestenbaum B., Hirsch I.B., Tuttle K.R., Himmelfarb J., de Boer I.H. (2013). Kidney disease and increased mortality risk in type 2 diabetes. J. Am. Soc. Nephrol..

[18] Cao J.J., Hudson M., Jankowski M., Whitehouse F., Weaver W.D. (2005). Relation of chronic and acute glycemic control on mortality in acute myocardial infarction with diabetes mellitus. Am. J. Cardiol..

[19] Marc C.C., Mot M.D., Licker M., Muntean D., Marti D.T., Ardelean A.A., Ciceu A., Sprintar S.A., Oatis D.A., Mihu A.G. (2025). Trends in positive urine culture rates and antimicrobial resistance in non-hospitalized children from western Romania: A retrospective observational study. Antibiotics.

[20] Hancı P., Cengizhan M.S., Yıldız Ç., İnal V. (2025). Glycemic variability and mortality in critically ill patients: Higher risk in non-diabetic patients. Turk. J. Intensive Care.

[21] Hsu C.-W., Lin C.-S., Chen S.-J., Lin S.-H., Lin C.-L., Kao C.-H. (2016). Risk of type 2 diabetes mellitus in patients with acute critical illness: A population-based cohort study. Intensive Care Med..

[22] Einarson T.R., Acs A., Ludwig C., Panton U.H. (2018). Prevalence of cardiovascular disease in type 2 diabetes: A systematic literature review of scientific evidence from across the world in 2007–2017. Cardiovasc. Diabetol..

[23] Galaviz K.I., Narayan K.M.V., Lobelo F., Weber M.B. (2018). Lifestyle and the prevention of type 2 diabetes: A status report. Am. J. Lifestyle Med..

[24] Gavelli F., Patrucco F., Bellan M. (2019). Diabetes and acute respiratory failure. Is the lung finally safe?. Int. J. Crit. Illn. Inj. Sci..

[25] Keryakos H.K.H., Hussein W.T., Abu-El-Ela M.A.E.-S., Helmy A.K. (2025). Predicting mortality in critically ill patients: A machine learning approach to electrolyte imbalances and clinical risk factors. J. Transl. Med..

[26] Villar J., González Martín J.M., Hernández-González J., Armengol M., Fernández C., Martín-Rodríguez C., Mosteiro F., Martínez D., Sánchez-Ballesteros J., Ferrando C. (2023). Predicting ICU mortality in acute respiratory distress syndrome patients using machine learning: The predicting outcome and stratification of severity in ARDS (POSTCARDS) study. Crit. Care Med..

[27] Pliszka M., Szablewski L. (2025). Severe insulin resistance syndromes: Clinical spectrum and management. Int. J. Mol. Sci..

[28] Liao T.I., Ho C.Y., Chin S.C., Wang Y.C., Chan K.C., Chen S.L. (2024). Sequential impact of diabetes mellitus on deep neck infections: Comparison of the clinical characteristics of patients with and without diabetes mellitus. Healthcare.

[29] Ma H., Yu G., Wang Z., Zhou P., Lv W. (2022). Association between dysglycemia and mortality by diabetes status and risk factors of dysglycemia in critically ill patients: A retrospective study. Acta Diabetol..

[30] Michalia M., Kompoti M., Koutsikou A., Paridou A., Giannopoulou P., Trikka-Graphakos E., Clouva-Molyvdas P. (2009). Diabetes mellitus is an independent risk factor for ICU-acquired bloodstream infections. Intensive Care Med..

[31] Zhao J., Huang D., Hua S., Huang X., Chen Y., Zhuang Y. (2025). Time in targeted blood glucose range as an independent predictor of 28-Day mortality in ICU patients: A retrospective study. Diabetes Res. Clin. Pract..

[32] Bondar A., Popa A.R., Papanas N., Popoviciu M., Vesa C.M., Sabau M., Daina C., Stoica R.A., Katsiki N., Stoian A.P. (2021). Diabetic neuropathy: A narrative review of risk factors, classification, screening and current pathogenic treatment options (Review). Exp. Ther. Med..

[33] Paduraru L., Zaha D.C., Ghitea T.C., Fodor R., Vesa C.M., Popoviciu M.S. (2025). Integrating renal and metabolic parameters into a derived risk score for hyperuricemia in uncontrolled type 2 diabetes: A retrospective cross-sectional study in northwest Romania. Medicina.

[34] Pasquel F.J., Lansang M.C., Dhatariya K., Umpierrez G.E. (2021). Management of diabetes and hyperglycaemia in the hospital. Lancet Diabetes Endocrinol..

[35] Vidger A.J., Czosnowski Q.A. (2021). Outcomes and adverse effects of extremely high dose insulin infusions in ICU patients. J. Crit. Care.

